# MRD-UNet: a compute-efficient medical image segmentation architecture with multi-attention and parallel dilated fusion

**DOI:** 10.1186/s12880-026-02680-x

**Published:** 2026-08-13

**Authors:** Musa Dogan, Ilker Ali Ozkan

**Affiliations:** Department of Computer Engineering, Faculty of Technology, Selcuk University, Konya, 42150 Türkiye

**Keywords:** Attention mechanisms, Dilated convolution, Medical image segmentation, MRD-UNet, Residual learning, U-Net variants

## Abstract

**Background:**

Medical image segmentation plays a critical role in computer-aided diagnosis by enabling precise delineation of abnormalities in medical images. Accurate segmentation of these images is essential for early diagnosis and treatment planning. However, conventional models such as U-Net face challenges including boundary uncertainty, variations in image quality and limited generalization across datasets.

**Methods:**

This study presents MRD-UNet, a U-Net based segmentation framework built on a channel-disjoint multi-path dilated fusion (MDF) block deployed uniformly across all encoder and decoder stages, a residual formulation within each MDF block that stabilizes gradient flow without introducing pooling branches, and a task-driven distribution of three functionally distinct attention modules. We evaluate the proposed model through extensive experiments on the CVC-ClinicDB dataset comprising 612 endoscopic images and the ISIC-2018 dataset containing 2,594 dermoscopic images. During data augmentation, we apply geometric transformations, elastic deformations, and brightness adjustments; training is performed using a composite loss that combines Dice loss and binary cross-entropy.

**Results:**

The proposed method achieves a DSC of 0.9140 on CVC-ClinicDB and 0.9106 on ISIC-2018, yielding an improvement of approximately 2–3% over the baseline U-Net architecture. Ablation studies confirm that the channel-disjoint MDF design reduces the number of parameters relative to the baseline while improving sensitivity to small and low-contrast lesions, and that the targeted attention layout enhances boundary delineation.

**Conclusions:**

MRD-UNet provides a practical balance between segmentation accuracy and computational efficiency. It outperforms baseline CNNs and performs comparably to heavier transformer-based models while using significantly fewer parameters. Cross-dataset evaluations demonstrate its ability to generalize within the same imaging modality. Notably, the lightweight MRD-UNet-S variant maintains competitive accuracy, showing the architecture’s suitability for resource-constrained medical image segmentation tasks.

**Clinical trial number:**

Not applicable.

## Background

Medical images have become an integral part of diagnostic and treatment procedures performed by physicians. These images can reveal morphological lesions and functional changes that provide strong evidence for clinical disease diagnosis [1, 2]. Accordingly, medical image analysis plays a critical role in these processes. Accurate segmentation of lesions in medical images is a key factor that supports physicians in clinical diagnosis and analysis. Medical image segmentation serves to localize pathologies, monitor disease progression, and assist diagnosis. Developing an efficient and accurate medical image segmentation method enables physicians to interpret medical images correctly, saves time associated with manual processing and enables improved disease detection [3]. However, obtaining highly accurate segmentation results remains challenging due to factors such as blurred structural boundaries in the image, low contrast, and heterogeneous textures. In addition, the diverse appearances, irregular sizes, and unpredictable locations of certain organs and lesions in medical images make medical image segmentation particularly difficult. Indeed, early studies in this field relied on algorithms based on manual feature extraction. Classical segmentation techniques based on shape, texture and color analysis such as Otsu thresholding [4], fuzzy sets [5] and active contour [6] have generally proved inadequate for detecting medical diseases and have lacked the accuracy and robustness required for widespread clinical use.

With the increased availability of large-scale medical image datasets, manual feature learning with traditional methods has become impractical, making it feasible to develop deep learning models such as convolutional neural networks (CNNs). In this field, fully convolutional networks (FCN) [7] established a foundational framework as one of the earliest deep learning approaches capable of end-to-end segmentation by classifying pixels. Building on this architecture, various models have been proposed that tailor and extend it to the specific needs of medical imaging. In particular, U-Net [8] has been a forerunner in medical image segmentation, outperforming traditional image processing methods in this domain. To further enhance segmentation performance, numerous U-Net based models have been proposed, including Attention U-Net [9], ResU-Net [10], V-Net [11] and U-Net++ [12].

Although convolution-based methods demonstrate superior performance compared to traditional approaches, they often suffer from limitations such as a large number of trainable parameters and redundant fusion of feature channels, as well as difficulty in learning non-local and long-range features due to the limited receptive field of the convolution kernel [13]. To address these shortcomings, enhanced attention mechanisms [14–16], convolutions with dilation (also known as atrous convolutions) [17, 18] and transformer-based architectures [19–21] have emerged, enabling models to capture long-range dependencies more effectively. For instance, DHAFormer [22] proposes a dual-channel hybrid attention mechanism combining global context and local lesion branches alongside a multi-scale channel fusion module, achieving strong polyp segmentation performance on CVC-ClinicDB and Kvasir-SEG. In addition, the training process has been further refined through improved loss functions [23, 24] and semi-supervised learning strategies. A recent example is IPA-CP [25], which employs iterative pseudo-labeling and adaptive copy-paste supervision to address the scarcity of labeled data in tumor segmentation. Alongside these training-level advances, next-generation network architectures such as KAN-based U-Net [26, 27], MaxVit U-Net [28] and SSM-based models U-Mamba [29] and VM-UNet [30] have achieved promising results in various computer vision tasks.

Even though these sophisticated models have made real progress in addressing core issues, they come with their own complications. One major problem is that transformer-based architectures are built with quadratic computational complexity, which leads to increased computational load and memory consumption. Consequently, these models are typically slower to train and require larger scale labeled datasets to produce meaningful results. These constraints have motivated researchers to develop alternative architectures that are both highly expressive and more computationally efficient and explainable. So there’s obviously a gap that needs to be filled with improved convolution based segmentation models. What we’re looking for are architectures that can run efficiently, extract features better than standard convolutions, model complex channel relationships effectively, and do all this while keeping their decision-making transparent and traceable.

In recent years, U-Net variants incorporating parallel branches and dilated convolutions to expand multi-scale context have found their place in the literature; however, a significant portion of these studies combine features obtained from parallel branches using fixed rules. These rigid fusion strategies can lead to irrelevant information accumulation across channels, feature dilution, and inter-scale inconsistencies because they do not consider the contextual importance levels of different channels. This situation particularly limits segmentation performance in low-contrast and complex-structured medical images. Therefore, the fundamental motivation of this study is to develop a fusion mechanism that dynamically weighs and learns the importance of information from different scales and channels, rather than combining features with static rules. In this study, we propose a U-Net–based architecture, termed MRD-UNet, to enhance U-Net’s feature extraction capacity and address its current limitations. In this architecture, we design a multi-path dilated fusion (MDF) block for the encoder and decoder that lightens the network and reduces the number of parameters. Using this block, we extract features through parallel paths from different channels via dilated convolutions. At the bottleneck and in the decoding stages, multi attention mechanisms reinforce long-range interactions with positional information. We design the entire network in a residual form to mitigate the vanishing gradient problem, a common issue in deep architectures. In this way, we strengthen the representation of long-range dependencies with a convolution-based, lightweight design without resorting to the quadratic complexity of transformer-based methods.

This work addresses key limitations in literature and offers the following contributions:

- We introduce a multi-path dilated fusion (MDF) module that integrates channel-partitioned parallel branches with distinct dilation rates and a learned 1 × 1 fusion projection, effectively enlarging the receptive field while preserving computational efficiency through channel splitting.
- We develop a targeted multi-level attention strategy that assigns CA to each decoder stage for directional spatial recalibration, CBAM to the bottleneck for joint channel-and-spatial refinement, and AG to each skip connection to suppress semantically irrelevant encoder features before fusion.
- We perform a quantitative analysis of the synergy among core components in the multi-path and multi-attention designs through comprehensive ablation studies.
- By integrating the MDF and hybrid attention modules into a residual U-Net backbone, we present an architecture that is lighter than the baseline U-Net in terms of total parameters yet achieves superior segmentation performance on two public medical image datasets and demonstrates competitive generalization on a third unseen dataset under cross-dataset evaluation without fine-tuning.

### Related works

Convolutional neural network-based deep learning methods tailored for medical image segmentation commonly rely on U-Net which comprises encoder and decoder layers. Through skip connections established at matching spatial scales, U-Net improves model accuracy and alleviates the vanishing gradient problem. This foundational architectural success of U-Net has inspired numerous derivatives aimed at further enhancing performance. Some of these works enrich U-Net’s backbone with more advanced architectural components, while others redesign existing components within the architecture. In this way, the core framework is preserved yet combined with modern deep learning techniques to set new standards in segmentation accuracy. For example, the UNet++ [12] architecture redesigns the pathways between encoder and decoder with nested and dense skip connections to narrow the semantic gap. Sun et al. [31], employed a ResNet-50 backbone as the encoder, removed the final downsampling stage, and introduced dilated convolutions in its place. MSU-Net [32] replaced standard convolutional blocks with multi-scale blocks containing parallel convolution layers, aiming to capture both local and global contextual features. MDU-Net [33] introduced multi-scale dense connections in the encoder, the decoder, and the skip connections between them. Recent lightweight architectures like CMUNeXt [34] have redesigned skip connections using group convolutions and large kernels to extract global context and maximize inference speed. However, while these advanced methods improve spatial blending, many existing approaches still integrate multi-scale information without adaptively weighting parallel scale representations, which can lead to feature dilution and scale mismatch. In contrast, MRD-UNet overcomes this limitation with an MDF block that incorporates a learnable fusion mechanism to dynamically weight and integrate features.

The increasing complexity of deep neural networks has rendered the vanishing-gradient problem a significant obstacle during training. To mitigate this issue, He et al. [35] introduced the deep residual architecture (ResNet), which employs residual blocks to enable the efficient training of much deeper networks. By facilitating the flow of gradients to the network’s deepest layers, residual connections prevent gradient attenuation and avert the performance degradation commonly observed as network depth grows. Consequently, residual learning has been widely adopted in segmentation-oriented architectures. Zhang et al. [10] proposed ResU-Net, replacing standard convolutional blocks with deep residual blocks to alleviate vanishing gradients while using fewer parameters. Another residual-based model, R2U-Net [36] combines residual and recurrent units and achieves superior results compared with U-Net. ResUNet++ [37] further integrates residual blocks with dense skip connections, enhancing the fusion of multi-scale features. Studies also exist that enhance the effectiveness of residual blocks with architectural components such as dilated convolutions. In the study named DRUNET [38] the authors aimed to improve gradient flow while more effectively capturing information such as the spatial arrangement of tissues by combining these two structures. In another study, the HDA-ResUNet [17] architecture builds on the residual U-Net structure by adding channel attention and hybrid dilated convolution modules. In this model, channel attention blocks placed in the skip connections emphasize important channels in the feature maps from the encoder, while the hybrid dilated attention channel layer used at the network’s bottom layer aims to increase the capacity to evaluate multi-scale context by combining information from different receptive fields. In the literature, the combined use of residual, dilated and attention mechanisms has been effective in improving segmentation performance. The MRD-UNet we propose in this study adopts this approach but aims to go beyond previous methods with learnable multi-path fusion and attention mechanisms strategically integrated at different levels.

Attention mechanisms, which provide a deeper perspective on inter-pixel dependencies in medical image segmentation, are effective constructs that enhance feature encoding by directing networks to critical information in feature maps [39]. Among these approaches, attention gates (AG) were originally inspired by text processing tasks in natural language processing and subsequently tailored specifically for medical image segmentation [9, 40]. These modules learn to focus on target structures of varying shapes and sizes without increasing computational overhead, suppressing feature activations in irrelevant regions while emphasizing informative features to improve model sensitivity. The attention gate module proposed by Oktay et al. [9] adopts a grid-based attention computation, augmenting the representation of target regions via element-wise multiplication between feature maps and attention coefficients. Another important development in attention mechanisms is the emergence of modules that combine both spatial and channel attention. In this domain, the CBAM [41] provides comprehensive feature calibration with spatial and channel attention to yield a stronger feature representation. A lighter alternative in the literature, coordinate attention (CA), was proposed to address the loss of positional information caused by 2D global pooling in conventional channel attention mechanisms [42]. In this method, pooling kernels with two different spatial extents, (H,1) and (1, W), are employed to encode channel responses separately along the horizontal and vertical directions for a given input tensor. This decomposition strategy captures long-range spatial dependencies along one direction while preserving precise positional information along the other, enabling more accurate localization of target objects.

Attention and transformer-based mechanisms have been integrated into the U-Net architecture, introducing innovations particularly in the encoder and along the skip connections. Fan et al. [43] proposed a component termed the parallel partial decoder (PPD) to aggregate high-level feature maps produced by the encoder. Coupled with a reverse attention module, their design suppresses previously segmented regions to emphasize boundary areas. In DCSAU-Net [15] the decoder layers employ a lightweight and efficient compact split-attention (CSA) block to process the concatenated features arriving through the skip connections. Several studies have instead focused on improving the skip connections between the encoder and decoder [16, 44, 45]. In this strategy, some works adopt a hybrid approach to better filter the most informative features, whereas others replace the skip connection block entirely with a transformer-based design. There are also studies that adopt a multi-attention paradigm across different layers of architecture. Rather than using a single attention pathway, CA-Net [46] integrates a comprehensive attention scheme comprising spatial, channel, and scale attention into multiple points of the network. TransUNet partitions convolution-derived feature maps into patches and feeds them to a transformer encoder at the deepest stage, thereby capturing global contextual information. Similarly, DA-TransUNet [44] employs a hybrid encoder, but combines the transformer module with attention mechanisms for more refined features. It also appends a dual attention block to each skip connection to effectively filter irrelevant information passed from the encoder to the decoder. In recent years, transformer-based methods have demonstrated strong performance by more effectively capturing global context. Nevertheless, these gains often come with substantial computational cost and increased model complexity. Rather than adopting global self-attention, MRD-UNet distributes complementary attention operations across encoder, skip connection, and decoder stages within a residual convolutional backbone, achieving competitive segmentation performance at substantially lower computational cost. The remainder of the paper is organized as follows: The Methods section presents the proposed MRD-UNet architecture, with particular emphasis on the MDF block and the hybrid attention mechanisms. The Results section describes experimental setup, datasets, ablation studies, and comparative performance against state-of-the-art models. The Discussion section evaluates the core findings, computational efficiency, and limitations of the study. Finally, the Conclusions section summarizes the main contributions and outlines directions for future research.

## Methods

The primary limitations in medical image segmentation include the significant variability of target objects in terms of size, shape, and texture, as well as ambiguous boundaries that are difficult to separate from the background or adjacent tissues. While the standard U-Net architecture provides partial solutions to these challenges through its encoder-decoder structure and skip connections, it remains limited in its ability to simultaneously process features across multiple scales. To overcome these limitations, we propose MRD-UNet, a segmentation framework that extends the feature extraction and fusion capabilities of U-Net through a multi-path architecture operating at different network depths. The framework is designed to improve multi-scale feature representation, refine semantic information, and enhance positional sensitivity while preserving spatial resolution throughout the network.

### Overall structure of MRD-UNet

**Fig. 1:**
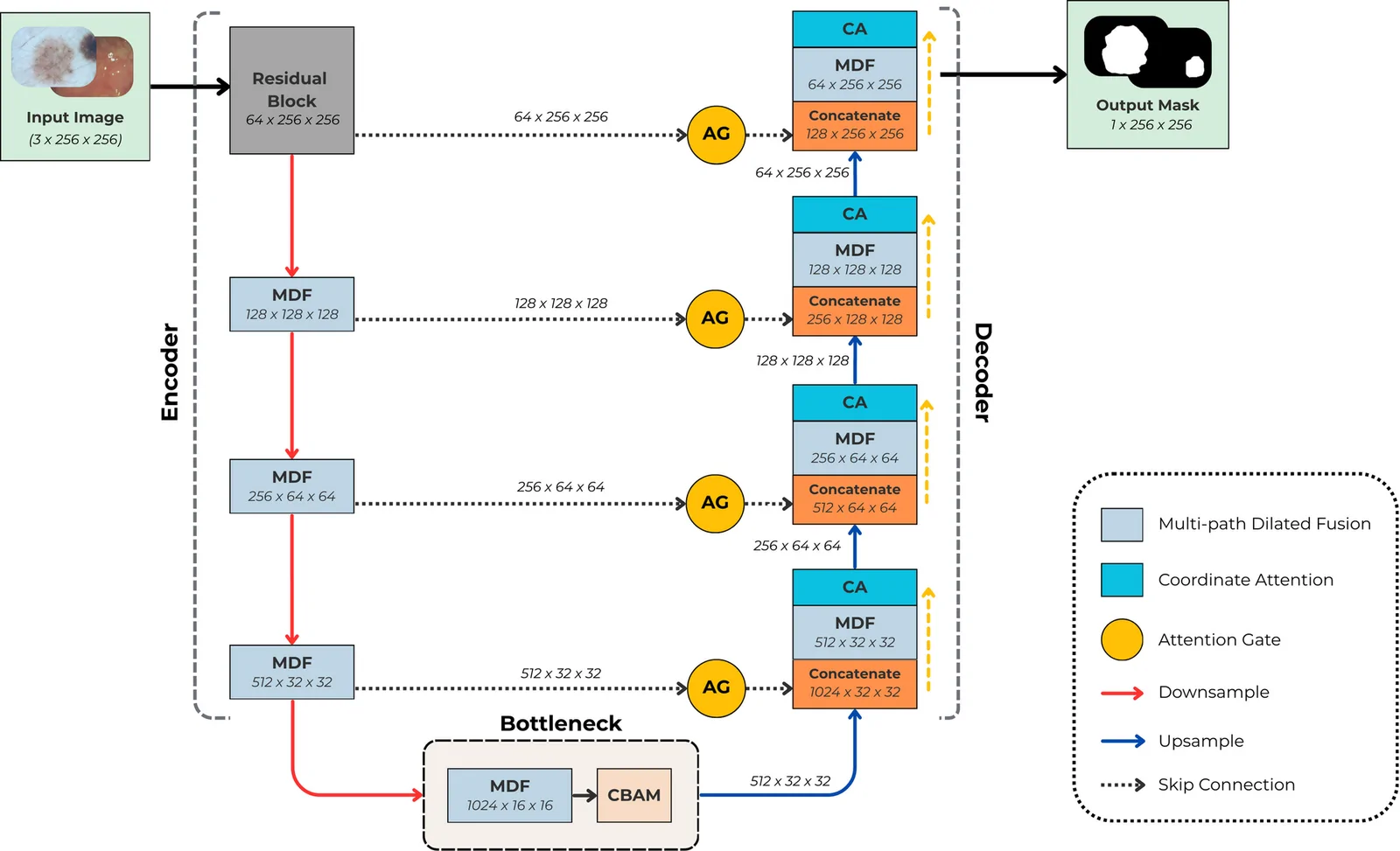
Structure of our proposed MRD-UNet architecture

The proposed MRD-UNet is a medical image segmentation network based on the encoder-decoder structure of the classic U-Net architecture, as illustrated in Fig. 1 and detailed layer-by-layer in Table 1. It processes input images through a symmetric encoder-decoder pathway in which spatial resolution is progressively reduced during encoding and restored during decoding. While the individual components of MRD-UNet draw on established techniques, the novelty of the architecture lies in three design decisions that have not been combined in this manner in prior work: the uniform deployment of MDF blocks across all encoder and decoder stages (rather than at a single bottleneck), the channel-disjoint branch structure that enforces representational complementarity within each block, and the targeted placement of three functionally distinct attention mechanisms at the specific locations where each addresses a different representational bottleneck.

**Table 1:** A layer-by-layer architecture of MRD-UNet

Stage	Input	Operation	Output	Stage	Input	Operation	Output
**Enc.**				**Dec.**			
Initial	3 × 256²	ResBlock	64 × 256²	D₁	1024 × 16²	UO + MDF + CA	512 × 32²
E₁	64 × 256²	MDF(s = 2)	128 × 128²	D₂	512 × 32²	UO + MDF + CA	256 × 64²
E₂	128 × 128²	MDF(s = 2)	256 × 64²	D₃	256 × 64²	UO + MDF + CA	128 × 128²
E₃	256 × 64²	MDF(s = 2)	512 × 32²	D₄	128 × 128²	UO + MDF + CA	64 × 256²
E₄	512 × 32²	MDF(s = 2) + CBAM	1024 × 16²	Final	64 × 256²	Conv1 × 1	1 × 256²

In the encoder, instead of standard convolution blocks, MDF blocks are used to extract features from multiple receptive fields in parallel and fuse them before downsampling. The input image is first processed by an initial residual convolution block to move it from pixel space to feature space. In the following layers, each MDF block extracts multi-scale features through parallel convolutional paths with distinct dilation rates; after each block, a 2 × 2 max-pooling operation halves the spatial resolution of the feature map while doubling the channel depth. This hierarchical and multi-scale feature extraction strategy enables the model to effectively capture both the ambiguous boundaries of lesions and the overall morphology of large lesions that span a wide area, simultaneously.

At the deepest level of the encoder, following an MDF block, we inserted a CBAM module sequentially refines the feature maps along the channel and spatial dimensions. Placing CBAM exclusively at the bridge is not arbitrary: at this depth, the representation operates at maximum semantic abstraction and minimum spatial resolution, making it the optimal location for global channel and spatial selection. Applying CBAM at earlier stages would risk suppressing spatially fine-grained features before they are consumed by skip connections.

In the decoder, the final segmentation map is progressively reconstructed by fusing low-resolution semantic features from the bottleneck with high-resolution spatial details from the encoder. At each decoder level, the feature map from the previous layer is upsampled using a 2 × 2 transposed convolution. At each skip connection, an attention gate suppresses irrelevant background activations in the encoder-derived feature maps conditioned on the decoder’s current semantic context, a function that is distinct from CBAM since CBAM operates within a single stream rather than across streams. Following the MDF block at each decoder stage, coordinate attention enhances directional spatial awareness along the horizontal and vertical axes independently, contributing to the precise reconstruction of lesion boundaries. Standard global average pooling-based attention collapses all spatial information into a scalar, losing directional structure. CA avoids this by pooling along each axis separately, and is therefore placed in the decoder where boundary reconstruction is the primary task.

As shown in Table 1, the initial ResBlock uses a 1 × 1 convolution block to project the 3 × 256² input into a 64 × 256² feature space. In stages E₁–E₄, MDF blocks with stride (s) = 2 extract features, halving the spatial resolution while doubling the number of feature channels. At the bottleneck (E₄), CBAM recalibrates the feature representation sequentially along channel and spatial dimensions before features are forwarded to the decoder. In stages D₁–D₄, following upsampling with a ConvTranspose2d-based up-sampling operation (UO), the corresponding encoder feature is filtered with an AG and the outputs are concatenated before an MDF block performs multi-scale fusion. In the decoder, MDF performs multi-scale fusion, and CA applies direction-aware spatial reweighting. This design preserves fine boundary details while suppressing task-irrelevant activations throughout the decoding path.

### Dilated convolution

Dilated convolution [47], first introduced as a conference paper at ICLR 2016 and also known in the literature as atrous convolution, is a technique used to geometrically expand the receptive field of the standard convolution operation without introducing additional parameters or computational cost. This method works by inserting gaps, controlled by a parameter called the dilation rate ($\:{d}_{n}$) between the elements of the convolution kernel. For a two-dimensional input map * x* and a kernel * w* the dilated convolution operation for each location (* i*) on the output map * y* is expressed as follows:

1 $$y\left[ i \right] = \mathop \sum \limits_k x\left[ {i + {d_n} \cdot k} \right] \cdot w\left[ k \right]$$

where, $\:{d}_{n}=\:1$ corresponds to standard convolution, while $\:{d}_{n}\:>\:1$ enables the kernel to skip pixels and aggregate information from a wider spatial extent without downsampling.

In medical image segmentation, this approach allows the model to analyze a broader contextual window while preserving spatial resolution, which is crucial for understanding a lesion’s relationship with surrounding tissues. However, stacking layers with the same dilation rate can lead to an artifact known as the gridding effect, which may cause the loss of local information from neighboring pixels. To address this gridding effect and to capture contextual information across scales in a balanced manner, MRD-UNet adopts the multi-path dilated fusion (MDF) approach detailed in the next section which integrates parallel convolutional branches with different dilation rates instead of relying on a single rate.

### Multi-path dilated fusion block

**Fig. 2:**
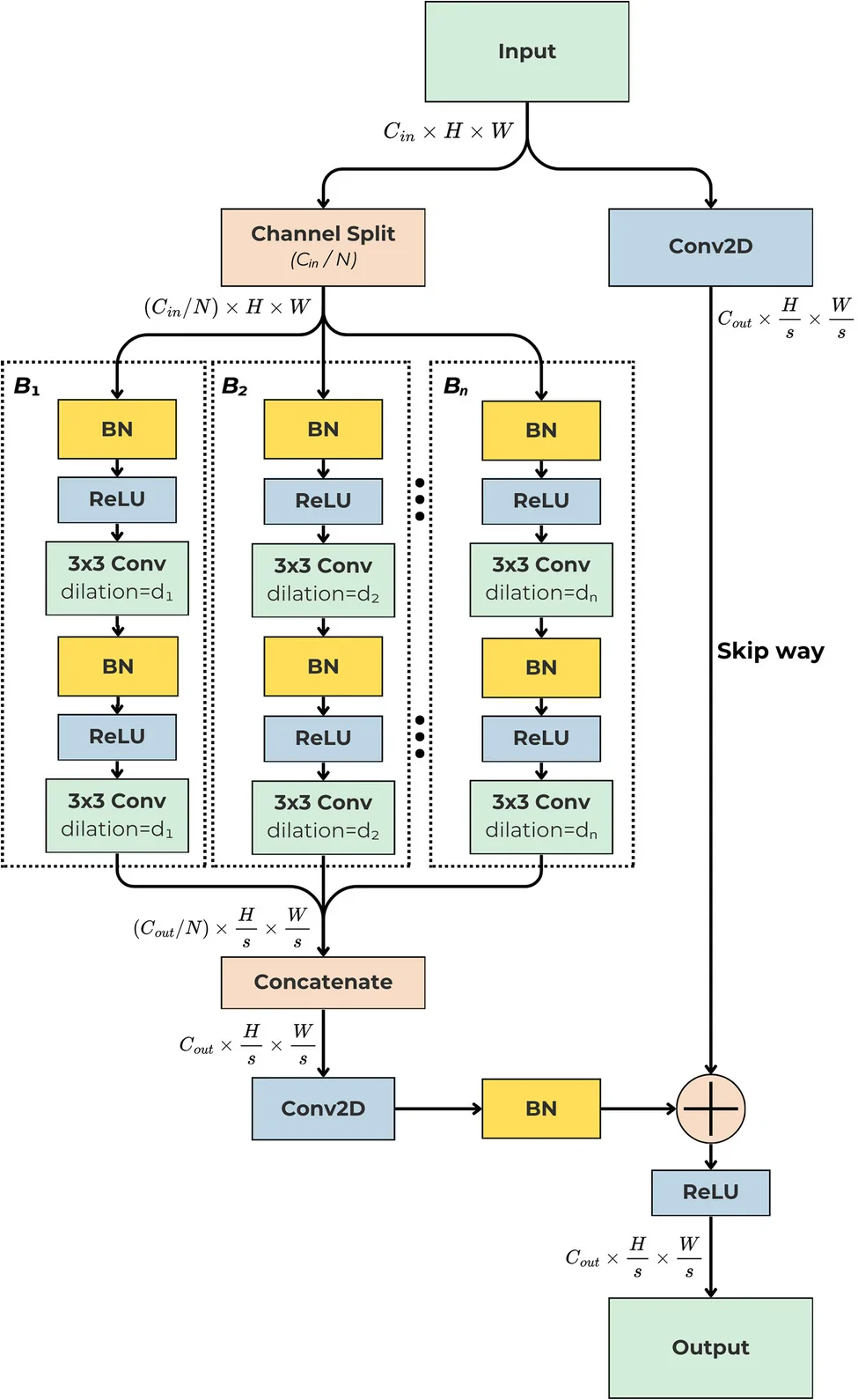
Overview of proposed MDF block

We designed the multi-path dilated fusion (MDF) block, the fundamental building unit of the MRD-UNet, to simultaneously address the gridding artifact, capture contextual information across multiple spatial scales, and facilitate gradient flow in deep networks through residual learning. As illustrated in Fig. 2, this block augments a standard residual block by incorporating parallel convolutional branches with different dilation rates. The input feature map$$\:\:X\in\:{\mathbb{R}}^{{C}_{in}\times\:H\times\:W}$$ is partitioned into * N* disjoint channel subsets $$\:X=\left[{X}_{1},{X}_{2},\dots\:,{X}_{N}\right]$$ where $$\:{X}_{n}\in\:{\mathbb{R}}^{\left(\raisebox{1ex}{${C}_{in}$}\!\left/\:\!\raisebox{-1ex}{$N$}\right.\right)\times\:H\times\:W}$$. Each subset is routed exclusively to a single branch and is never shared with other branches, unlike ASPP [48] and Inception-style modules [49], which route the full feature map to every branch, allowing redundant responses across branches. Each branch * n* applies two consecutive 3 × 3 dilated convolutions with dilation rate $$\:{d}_{n}$$, each preceded by batch normalization (BN) and ReLU activation, producing output $$\:{B}_{n}$$. The branch outputs are concatenated along the channel dimension, fused with a 1 × 1 convolution, and normalized with batch normalization. A skip connection then adds the projected input directly to the fused output, and a final ReLU yields the block output $$\:Y=ReLU\left(F+{W}_{s}\cdot\:X\right)$$, enabling residual learning. Unlike ASPP, which applies fixed dilation rates (6, 12, 18) at a single bottleneck location, MRD-UNet assigns distinct dilation rate pairs to each MDF block in a layer-adaptive manner: early encoder stages with high spatial resolution receive smaller rate pairs (e.g., $$\:\left\{\mathrm{1,3}\right\}$$) to preserve fine-grained local detail, while intermediate stages receive wider rates (e.g., $$\:\left\{\mathrm{2,5}\right\}$$) to capture broader context. The path with $$\:{d}_{n}$$ =1 behaves like a standard convolution, capturing fine-grained, high-frequency details, while paths with $$\:{d}_{n}$$ > 1 aggregate global contextual information such as the shape and structure of large objects. The selection of these specific rate pairs was guided by two principles: avoiding the gridding effect by ensuring no two consecutive layers share identical rates, and empirical validation through a systematic sensitivity analysis (Table 9). Furthermore, neither Inception nor ASPP incorporates a residual connection, while MDF includes an explicit skip connection; both also contain a pooling branch (MaxPool and Global Average Pooling, respectively), whereas MDF contains no pooling branch, relying solely on dilated convolutions with expanding receptive fields.

MDF blocks are placed in every encoder stage, in the bridge, and in each decoder stage after skip-connection fusion. The consistent use of MDF blocks along both the encoder and decoder paths ensures that features are processed from a multi-scale perspective at every stage; the ablation studies in the subsequent section quantify this block’s contribution and further confirm that the multi-path architecture outperforms a single-rate dilated residual block, which is the configuration that would most closely resemble an ASPP-style design.

### Coordinate attention

**Fig. 3:**
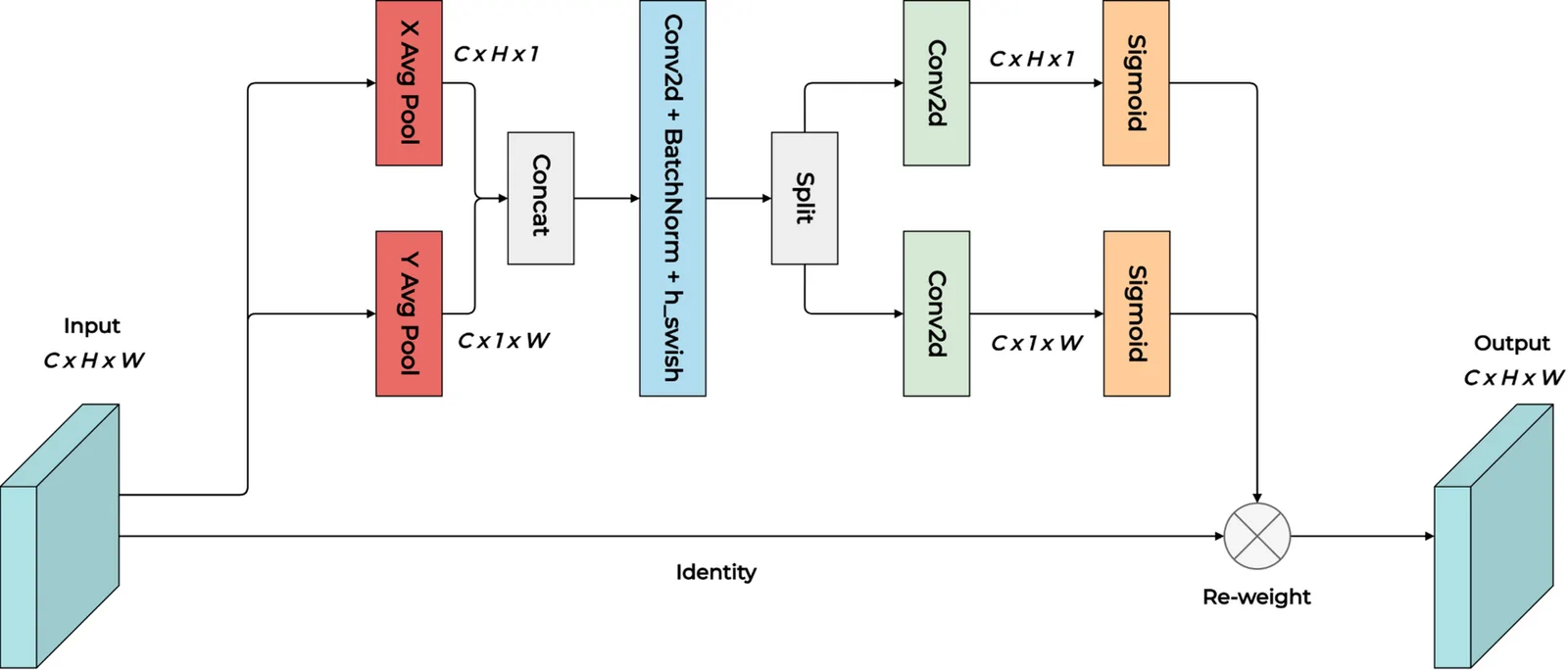
Schematic diagram of coordinate attention

The coordinate attention (CA) mechanism comprises two main steps: coordinate information embedding and coordinate attention generation. In the first step, unlike conventional channel attention that compresses all spatial information into a single value via global average pooling, CA decomposes this operation into two one-dimensional encodings. The input feature map is average pooled separately along the horizontal (X) and vertical (Y) axes. The resulting two tensors capture long-range dependencies in one direction while preserving precise positional information in the orthogonal direction. In the second step, these two direction-aware features are concatenated and passed through a 1 × 1 convolution, followed by batch normalization and the h-swish activation function, to model their interdependence. The fused features are then split back into horizontal and vertical components; separate 1 × 1 convolutions and sigmoid activations produce the horizontal and vertical attention weights. Finally, these attention maps reweight the original input via element-wise multiplication to yield the output. This process enables the model to focus not only on salient channels but also on the exact spatial locations of objects within those channels, which improves segmentation quality in medical images with complex and ambiguous boundaries. As shown schematically in Fig. 3, we apply CA in the decoder stages after multi-scale fusion to reweight features both channel-wise and with direction-aware spatial context, thereby enhancing the localization of fine boundaries and small lesions.

## Results

In this section, we present the experimental studies conducted to validate the performance of MRD-UNet. We first describe the datasets used, followed by the implementation details and training procedure. We then provide the evaluation metrics and finally discuss the comparisons with SOTA methods and the ablation analyses.

### Dataset

To evaluate our model’s generalization across different medical domains and its robustness under challenging conditions, we selected three distinct and widely used public datasets. The first is the ISIC-2018 Challenge [50, 51] dataset, which is frequently employed in medical segmentation studies. In this work, we used the lesion segmentation (Task 1) from this dataset. It contains 2,594 dermoscopic images of skin lesions acquired from real patients using a digital camera, along with their ground-truth masks. The annotations were generated using several techniques and reviewed by experienced dermatologists. Notably, the ISIC-2018 images have variable, non-uniform resolutions. The second dataset is CVC-ClinicDB [52] which comprises 612 frames extracted from colonoscopy videos and their corresponding ground-truth masks, commonly used for colorectal polyp segmentation and detection. Each image has a resolution of 384 × 288 pixels. The third dataset is PH2 [53], a dermoscopic image database developed at the Pedro Hispano Hospital (Matosinhos, Portugal) under the same acquisition conditions using a Tuebinger Mole Analyzer system. It consists of 200 dermoscopic images of melanocytic lesions (80 common nevi, 80 atypical nevi, and 40 melanomas) along with corresponding ground-truth segmentation masks annotated by expert dermatologists. Each image has a resolution of 768 × 560 pixels. In this study, PH2 is used exclusively for cross-dataset evaluation: all models are trained on ISIC-2018 and tested directly on PH2 without any fine-tuning, in order to assess generalization to unseen dermoscopic data.

### Implementation

In this study, all experimental procedures were conducted on an NVIDIA Quadro RTX 8000 using the PyTorch deep learning framework. For both datasets, the batch size was set to 16, and input images were resized to a resolution of 256 × 256 pixels. After preliminary trials, to prevent overfitting, we trained each model for 200 epochs on the CVC-ClinicDB dataset and 100 epochs on the ISIC-2018 dataset. Given the limited amount of annotated data, we employed data augmentation during training using Albumentations [54]. The augmentations included random rotation, horizontal and vertical flipping, elastic transformation, grid distortion, brightness–contrast adjustment, color jitter, and various blurring operations. The CVC-ClinicDB dataset was split into 70% training, 15% validation, and 15% test; the ISIC-2018 dataset was split into 80% training, 10% validation, and 10% test. To ensure reproducibility, datasets were randomly partitioned according to these ratios using a fixed random seed. All results are reported as the mean across three random seeds. To maintain consistency of the reported results, we applied the same hyperparameters across all model trainings. Hyperparameters were optimized with reference to pilot experiments on the validation set and to comparable studies in literature. For model optimization, we used the AdamW optimizer [55], with an initial learning rate of 0.0001. A OneCycleLR scheduler was employed to increase the learning rate up to a maximum of 0.001. The tumor region is often substantially smaller than the background, introducing class imbalance. To address this, we combined Dice loss and binary cross-entropy (BCE) loss as the training objective.

2 $$\:{\mathcal{L}}_{sum}\:=\:\alpha\:\:.{\mathcal{L}}_{BCE}\:+\:{\beta\:\mathcal{\:}.\mathcal{\:}\mathcal{L}}_{Dice}$$

where, the weighting constants $\:\alpha\:$ and $\:\beta\:$ are set to 1 following established practice in medical image segmentation [56, 57]. We can expand the $\:{\mathcal{L}}_{sum}$ formula as follows:

3 $${{\cal L}_{BCE}} = - {1 \over n}\mathop \sum \limits_{i = 1}^n \left( {{Y_i} \cdot {\rm{log}}{{\hat Y}_i} + \left( {1 - {Y_i}} \right) \cdot {\rm{log}}\left( {1 - {{\hat Y}_i}} \right)} \right)$$

4 $${{\cal L}_{Dice}} = 1 - {{2\sum\limits_{i = 1}^n {{Y_i}{{\hat Y}_i} + \epsilon } } \over {\sum\limits_{i = 1}^n {{Y_i} + } \sum\limits_{i = 1}^n {{{\hat Y}_i} + \epsilon } }}$$

where $\:{Y}_{i}\in\:\left\{\mathrm{0,1}\right\}$ is the ground truth label of the * i*-th pixel, $\:{\widehat{Y}}_{i}\in\:\left[\mathrm{0,1}\right]$ is the corresponding predicted probability, * n* is the total number of pixels, and $\epsilon={10}^{-6}$ is a smoothing constant to prevent division by zero.

### Evaluation metrics

In the experiments, we used five metrics: dice similarity coefficient (DSC), intersection over union (IoU), recall, precision and 95th percentile Hausdorff distance (HD95) to assess segmentation quality more holistically across all models. These metrics are provided in Eqs. 5–9, respectively. In the formulas, TP denotes the number of true positives, FP false positives, FN false negatives, and TN true negatives. DSC, which measures the overlap between the predicted mask and the reference (ground-truth) mask, is adopted as the primary performance measure. Higher mean scores of DSC and the other metrics indicate better network performance. All metric values are reported in mean±standard deviation (µ ± σ) format.

5 $${\rm{DSC = }}{{{\rm{2}} \cdot {\rm{TP}}} \over {{\rm{2}} \cdot {\rm{TP + FP + FN}}}}$$

6 $$\:\mathrm{IoU}\mathrm{=}\frac{\mathrm{TP}}{\mathrm{TP}\text{}\mathrm{+}\mathrm{FP}\mathrm{+}\mathrm{FN}}$$

7 $$\:\mathrm{Recall}\mathrm{=}\frac{\mathrm{TP}}{\mathrm{TP}\text{}\mathrm{+}\mathrm{FN}}$$

8 $$\:\mathrm{Precision}\mathrm{=}\frac{\mathrm{TP}}{\mathrm{TP}\mathrm{+FP}}$$

9 $$\:\mathrm{H}\mathrm{D}95=\mathrm{max}\left({d}_{95\left(P\to\:G\right)},{d}_{95\left(G\to\:P\right)}\right)$$

where $\:{d}_{95}(P\to\:G)$ denotes the 95th percentile of distances from predicted boundary * P* to ground-truth boundary * G*, and $\:{d}_{95}(G\to\:P)$ denotes the same in the reverse direction. Unlike other metrics, HD95 has no fixed upper bound; lower values indicate better boundary localization.

We evaluate the performance of the proposed MRD-UNet architecture through comprehensive experimental analyses. The study presents quantitative comparisons against baseline and state-of-the-art methods on CVC-ClinicDB and ISIC-2018, followed by cross-dataset generalization experiments on PH2 to assess model robustness without fine-tuning. We then provide computational efficiency comparisons including FPS, GFLOPs, and parameter counts evaluated on the same hardware, alongside a parameter-efficient variant, MRD-UNet-S. Subsequently, ablation studies isolate the contribution of each architectural component, and a dilation rate sensitivity analysis justifies the selected MDF configuration. Finally, qualitative analyses on challenging cases complement the quantitative findings.

### Quantitative comparison with baseline networks and SOTA models

Tables 2 and 3 present the quantitative results on CVC-ClinicDB and ISIC-2018, respectively. On CVC-ClinicDB (Table 2), MRD-UNet achieves a DSC of 0.9140 and mIoU of 0.8596, outperforming all baseline models: +2.23% DSC over UNet (0.8917), + 3.08% over AttUNet (0.8832), + 4.43% over ResUNet (0.8697), and + 2.13% over UNet++ (0.8927). Among recent methods, TransUNet (0.9285) and PraNet (0.9223) yield higher DSC scores on this dataset, while MRD-UNet outperforms CMUNeXt (0.8974) and U-Mamba (0.9013), and remains comparable to DA-TransUNet (0.9190). The parameter and throughput trade-offs of these methods are examined separately in Table 4. On ISIC-2018 (Table 3), MRD-UNet achieves a DSC of 0.9106 ± 0.0023 and mIoU of 0.8479 ± 0.0029, outperforming all compared methods including PraNet (0.9101), TransUNet (0.9079), UNet++ (0.9062), and U-Mamba (0.9067). HD95 of 3.61 ± 0.19 is also the lowest among all models on this dataset. MRD-UNet-S achieves a DSC of 0.9007 on CVC-ClinicDB and 0.9101 on ISIC-2018 at approximately 118 FPS, representing a 74% throughput increase over MRD-UNet with a 1.33% and 0.05% reduction in DSC, respectively. Wilcoxon signed-rank tests indicate that improvements over UNet, AttUNet, ResUNet, and U-Mamba are statistically significant on both datasets (*p* < 0.05). On CVC-ClinicDB, MRD-UNet achieves competitive performance relative to TransUNet, with no statistically significant difference between the two models (*p* = 0.156); on ISIC-2018, MRD-UNet outperforms TransUNet with statistical significance (*p* = 0.011). Improvements over UNet + + are significant on CVC-ClinicDB (*p* = 0.026) but do not reach significance on ISIC-2018.

**Table 2:** Comparison of MRD-UNet with baseline and SOTA models on the **CVC-ClinicDB** dataset

Networks	DSC↑	mIoU↑	Recall↑	Precision↑	HD95↓	FPS↑
UNet	0.8917 ± 0.0098	0.8302 ± 0.0119	0.8947 ± 0.0029	0.9250 ± 0.0138	10.14 ± 4.03	97.56
ResUNet	0.8697 ± 0.0073	0.7940 ± 0.0083	0.8667 ± 0.0087	0.9020 ± 0.0058	15.13 ± 2.49	115.79
AttUNet	0.8832 ± 0.0005	0.8198 ± 0.0016	0.8829 ± 0.0037	0.9227 ± 0.0015	13.91 ± 0.55	90.5
UNet++	0.8927 ± 0.0017	0.8341 ± 0.0029	0.8971 ± 0.0051	0.9266 ± 0.0086	12.15 ± 0.64	40.03
PraNet	0.9223 ± 0.0085	0.8723 ± 0.0091	0.9340 ± 0.0112	0.9284 ± 0.0036	6.51 ± 0.32	46.18
TransUNet	0.9285 ± 0.0026	0.8789 ± 0.0049	0.9251 ± 0.0026	0.9382 ± 0.0077	3.01 ± 0.47	49.91
CMUNeXt	0.8974 ± 0.0056	0.8410 ± 0.0055	0.9026 ± 0.0086	0.9257 ± 0.0052	10.95 ± 1.09	197.23
DA-TransUNet	0.9190 ± 0.0039	0.8666 ± 0.0030	0.9126 ± 0.0097	0.9422 ± 0.0081	6.29 ± 1.57	53.21
U-Mamba	0.9013 ± 0.0121	0.8400 ± 0.0172	0.9011 ± 0.0094	0.9196 ± 0.0120	7.76 ± 2.11	97.94
MRD-UNet-S	0.9007 ± 0.0085	0.8445 ± 0.0107	0.9022 ± 0.0112	0.9260 ± 0.0068	7.96 ± 1.19	117.55
MRD-UNet	0.9140 ± 0.0056	0.8596 ± 0.0066	0.9137 ± 0.0057	0.9308 ± 0.0075	5.14 ± 1.53	68.48

**Table 3:** Comparison of MRD-UNet with baseline and SOTA models on the **ISIC-2018** dataset

Networks	DSC↑	mIoU↑	Recall↑	Precision↑	HD95↓	FPS↑
UNet	0.9038 ± 0.0018	0.8383 ± 0.0025	0.9065 ± 0.0134	0.9274 ± 0.0124	4.30 ± 0.15	94.88
ResUNet	0.8795 ± 0.0019	0.8079 ± 0.0018	0.8918 ± 0.0049	0.9015 ± 0.0035	8.49 ± 0.46	113.24
AttUNet	0.9047 ± 0.0008	0.8396 ± 0.0008	0.9093 ± 0.0056	0.9260 ± 0.0062	4.34 ± 0.25	90.35
UNet++	0.9062 ± 0.0013	0.8407 ± 0.0015	0.9128 ± 0.0056	0.9234 ± 0.0050	4.38 ± 0.17	38.56
PraNet	0.9101 ± 0.0044	0.8457 ± 0.0057	0.9227 ± 0.0051	0.9187 ± 0.0082	3.47 ± 0.40	51.74
TransUNet	0.9079 ± 0.0032	0.8429 ± 0.0037	0.9155 ± 0.0077	0.9229 ± 0.0096	3.83 ± 0.39	58.56
CMUNeXt	0.9077 ± 0.0037	0.8438 ± 0.0043	0.9149 ± 0.0053	0.9237 ± 0.0064	4.29 ± 0.20	183.65
DA-TransUNet	0.9047 ± 0.0034	0.8404 ± 0.0042	0.9085 ± 0.0038	0.9281 ± 0.0057	4.08 ± 0.34	49.11
U-Mamba	0.9067 ± 0.0021	0.8440 ± 0.0025	0.9112 ± 0.0064	0.9285 ± 0.0082	3.96 ± 0.40	89.39
MRD-UNet-S	0.9101 ± 0.0022	0.8471 ± 0.0033	0.9155 ± 0.0073	0.9274 ± 0.0039	3.77 ± 0.10	117.76
MRD-UNet	0.9106 ± 0.0023	0.8479 ± 0.0029	0.9174 ± 0.0141	0.9263 ± 0.0121	3.61 ± 0.19	68.86

**Table 4:** Computational efficiency comparison. All models evaluated at 256 × 256 resolution on the same GPU

Model	Params (M) ↓	GFLOPs↓	Size (MB) ↓	CVC-ClinicDB		ISIC-2018		PH2	
				FPS↑	DSC↑	FPS↑	DSC↑	FPS↑	DSC↑
CMUNeXt	3.15	7.32	12.07	197.23	0.8974	183.65	0.9077	189.94	0.9080
**MRD-UNet-S**	5.20	8.20	19.88	117.55	0.9007	117.76	0.9101	110.91	0.9146
ResUNet	8.05	38.96	30.71	115.79	0.8697	113.24	0.8795	113.71	0.8718
U-Mamba	16.01	23.80	61.07	97.94	0.9013	89.39	0.9067	94.55	0.9143
**MRD-UNet**	20.76	32.51	79.30	68.48	0.9140	68.82	0.9106	69.08	0.9136
UNet	31.04	48.23	118.44	97.56	0.8917	94.88	0.9038	95.86	0.9038
AttUNet	31.39	49.33	119.79	90.50	0.8832	90.35	0.9047	90.49	0.9038
PraNet	32.55	6.92	124.41	46.18	0.9223	51.74	0.9101	56.09	0.9060
UNet++	36.62	138.17	139.76	40.03	0.8927	38.56	0.9062	38.83	0.9009
TransUNet	105.32	38.53	401.78	49.91	0.9285	58.56	0.9079	63.30	0.9119
DA-TransUNet	108.00	39.74	411.99	53.21	0.9190	49.11	0.9047	52.51	0.9120

### Computational efficiency analysis

Table 4 presents a comparison of computational efficiency across all evaluated models, including parameter count, GFLOPs, model size, and inference speed (FPS) measured at 256 × 256 resolution on the same GPU. Models are sorted by parameter count to facilitate comparison. MRD-UNet contains 20.76 M parameters and 32.51 GFLOPs, placing it between lightweight models such as CMUNeXt and larger transformer-based architectures such as TransUNet. It achieves 68.48 FPS on CVC-ClinicDB and 68.82 FPS on ISIC-2018, which is higher than TransUNet (49.91 / 58.56 FPS) and DA-TransUNet (53.21 / 49.11 FPS) despite comparable GFLOPs. TransUNet requires 105.32 M parameters, which is 5.1× more than MRD-UNet, while DA-TransUNet requires 108.00 M. Among lightweight models, CMUNeXt achieves the highest FPS (197.23 / 183.65) with only 3.15 M parameters, but yields lower DSC on both datasets (0.8974 / 0.9077). MRD-UNet-S contains 5.20 M parameters and 8.20 GFLOPs, achieving 117.55 FPS on CVC-ClinicDB and 117.76 FPS on ISIC-2018. This represents a 74% throughput increase over MRD-UNet, with DSC scores of 0.9007 and 0.9101 on the respective datasets. On PH2, MRD-UNet-S achieves the highest DSC among all evaluated models (0.9146) at 110.91 FPS.

### Ablation study

To assess the contribution of each architectural component, ablation experiments are conducted on both CVC-ClinicDB and ISIC-2018. All variants are trained and evaluated under identical conditions, with only the target component disabled and results are presented in Tables 5, 6, 7 and 8. Tables 5 and 6 examine the effect of attention mechanisms. Removing the attention gate (w/o AG) reduces DSC from 0.9200 to 0.9138 on CVC-ClinicDB and from 0.9128 to 0.9068 on ISIC-2018, indicating that attention-guided skip connections contribute to feature selectivity. Removing the coordinate attention block (w/o CA) leads to a more pronounced drop on CVC-ClinicDB (0.9066). Removing CBAM yields mixed results: DSC slightly increases on CVC-ClinicDB (0.9203) while decreasing on ISIC-2018 (0.9079). Tables 7 and 8 examine the contribution of the MDF block in the encoder and decoder separately. On CVC-ClinicDB, using MDF in both encoder and decoder achieves the highest DSC (0.9200), while removing the decoder MDF reduces DSC to 0.9146 and removing the encoder MDF leads to a larger drop (0.8863). On ISIC-2018, the differences are smaller in magnitude but follow a consistent pattern, with the full configuration (DSC = 0.9128) outperforming encoder-only (0.9100) and decoder-only (0.9063) variants.

**Table 5:** Ablation study on **CVC-ClinicDB**

Networks	DSC↑	mIoU↑	Recall↑	Precision↑	HD95↓	Params(M) ↓	GFLOPs↓	FPS↑
MRD-UNet (Proposed)	0.9200	0.8673	0.9122	0.9392	3.41	20.76	32.5	69.16
w/o AG	0.9138	0.8583	0.9178	0.9319	6.11	20.41	31.4	74.56
w/o CA	0.9066	0.8521	0.9036	0.9317	6.68	20.69	32.5	79.54
w/o CBAM	0.9203	0.8664	0.9156	0.9347	3.77	20.50	32.5	69.31

**Table 6:** Ablation study on **ISIC-2018**

Networks	DSC↑	mIoU↑	Recall↑	Precision↑	HD95↓	Params(M) ↓	GFLOPs↓	FPS↑
MRD-UNet (Proposed)	0.9128	0.8504	0.9193	0.9268	3.40	20.76	32.5	68.86
w/o AG	0.9068	0.8425	0.9164	0.9221	3.75	20.41	31.4	74.45
w/o CA	0.9084	0.8450	0.9110	0.9294	3.74	20.69	32.5	76.25
w/o CBAM	0.9079	0.8446	0.9178	0.9225	3.76	20.50	32.5	68.93

**Table 7:** Performance results of the ablation study for the MDF block integrated into an encoder-decoder architecture on the **CVC-ClinicDB** dataset

ID	Encoder	Decoder	DSC↑	mIoU↑	Recall↑	Precision↑	HD95↓	Params(M) ↓	GFLOPs↓	FPS↑
1	√	√	0.9200	0.8673	0.9122	0.9392	3.41	20.76	32.5	69.16
2	√		0.9146	0.8599	0.9217	0.9191	8.91	25.11	45.9	69.65
3		√	0.8863	0.8252	0.8797	0.9331	8.69	25.87	46.8	62.37
4			0.8858	0.8193	0.9098	0.8995	10.00	29.44	51.1	69.97

**Table 8:** Performance results of the ablation study for the MDF block integrated into an encoder-decoder architecture on the **ISIC-2018** dataset

ID	Encoder	Decoder	DSC↑	mIoU↑	Recall↑	Precision↑	HD95↓	Params(M) ↓	GFLOPs↓	FPS↑
1	√	√	0.9128	0.8504	0.9193	0.9268	3.40	20.76	32.5	68.86
2	√		0.9100	0.8468	0.9174	0.9251	3.63	25.11	45.9	69.14
3		√	0.9063	0.8416	0.9142	0.9229	3.96	25.87	46.8	61.99
4			0.9074	0.8439	0.9223	0.9177	3.80	29.44	51.1	69.47

### Hyperparameter sensitivity analysis

To justify the dilation rate selection in the MDF block, four configurations are evaluated on both datasets under identical architectural settings, varying only the per-layer dilation rate assignments. Results are presented in Table 9. The proposed default schedule achieves the highest DSC on both datasets. Configurations applying identical rates across all layers yield lower DSC on CVC-ClinicDB, suggesting that layer-wise progressive rate assignment captures multi-scale context more effectively than fixed uniform rates. The aggressive configuration produces the lowest DSC on both datasets, indicating that excessively large receptive fields are not beneficial at the spatial scales present in these tasks. These results indicate that the default progressive schedule provides a stable performance profile across both datasets without incurring additional computational cost.

**Table 9:** Sensitivity analysis of MDF dilation-rate configurations on CVC-ClinicDB and ISIC-2018

Dilation rates (enc = dec)	CVC-ClinicDB			ISIC-2018		
	DSC↑	IoU↑	HD95↓	DSC↑	IoU↑	HD95↓
**(1,3**→**2,5**→**2,5**→**1,3) (Default)**	0.9200	0.8673	3.41	0.9128	0.8504	3.40
(1,2→1,2→1,2→1,2)	0.9098	0.8484	6.39	0.9106	0.8475	3.60
(1,3→1,3→1,3→1,3)	0.9133	0.8581	7.24	0.9125	0.8503	3.17
(1,5→2,7→3,9→1,5)	0.9044	0.8484	7.42	0.9092	0.8466	4.05

### Cross dataset generalization

To examine whether the model maintains its performance on unseen data, all models trained on ISIC-2018 are evaluated directly on the PH2 dataset without any fine-tuning. Results are presented in Table 10. MRD-UNet achieves a DSC of 0.9136 on PH2, which is comparable to the best-performing methods TransUNet (0.9119) and DA-TransUNet (0.9120), and higher than UNet (0.9038), ResUNet (0.8718), AttUNet (0.9038), and UNet++ (0.9009). MRD-UNet-S achieves a DSC of 0.9146, the highest among all evaluated models on this dataset, at 110.91 FPS.

**Table 10:** Cross-dataset generalization: models trained on ISIC-2018 and tested on PH2 without fine-tuning

Networks	DSC↑	mIoU↑	Recall↑	Precision↑	HD95↓	FPS↑
UNet	0.9038 ± 0.0112	0.8334 ± 0.0166	0.9506 ± 0.0066	0.8788 ± 0.0167	11.73 ± 2.40	95.86
ResUNet	0.8718 ± 0.0057	0.7898 ± 0.0063	0.9018 ± 0.0113	0.8781 ± 0.0035	16.08 ± 0.49	113.71
AttUNet	0.9038 ± 0.0014	0.8323 ± 0.0022	0.9533 ± 0.0097	0.8750 ± 0.0085	11.61 ± 0.79	90.49
UNet++	0.9009 ± 0.0013	0.8285 ± 0.0036	0.9509 ± 0.0061	0.8737 ± 0.0097	12.79 ± 0.29	38.83
PraNet	0.9060 ± 0.0037	0.8347 ± 0.0060	0.9735 ± 0.0013	0.8587 ± 0.0072	9.67 ± 1.18	56.09
TransUNet	0.9119 ± 0.0073	0.8451 ± 0.0111	0.9704 ± 0.0036	0.8724 ± 0.0100	9.84 ± 2.68	63.30
CMUNeXt	0.9080 ± 0.0023	0.8405 ± 0.0041	0.9649 ± 0.0014	0.8727 ± 0.0057	10.74 ± 0.79	189.94
DA-TransUNet	0.9120 ± 0.0030	0.8461 ± 0.0044	0.9716 ± 0.0030	0.8724 ± 0.0052	9.29 ± 0.42	52.51
U-Mamba	0.9143 ± 0.0085	0.8484 ± 0.0138	0.9709 ± 0.0077	0.8746 ± 0.0167	8.35 ± 1.49	94.55
MRD-UNet-S	0.9146 ± 0.0037	0.8492 ± 0.0063	0.9677 ± 0.0091	0.8787 ± 0.0125	9.05 ± 0.76	110.91
MRD-UNet	0.9136 ± 0.0061	0.8476 ± 0.0095	0.9711 ± 0.0023	0.8736 ± 0.0120	8.80 ± 0.94	69.08

### Qualitative analysis

**Fig. 4:**
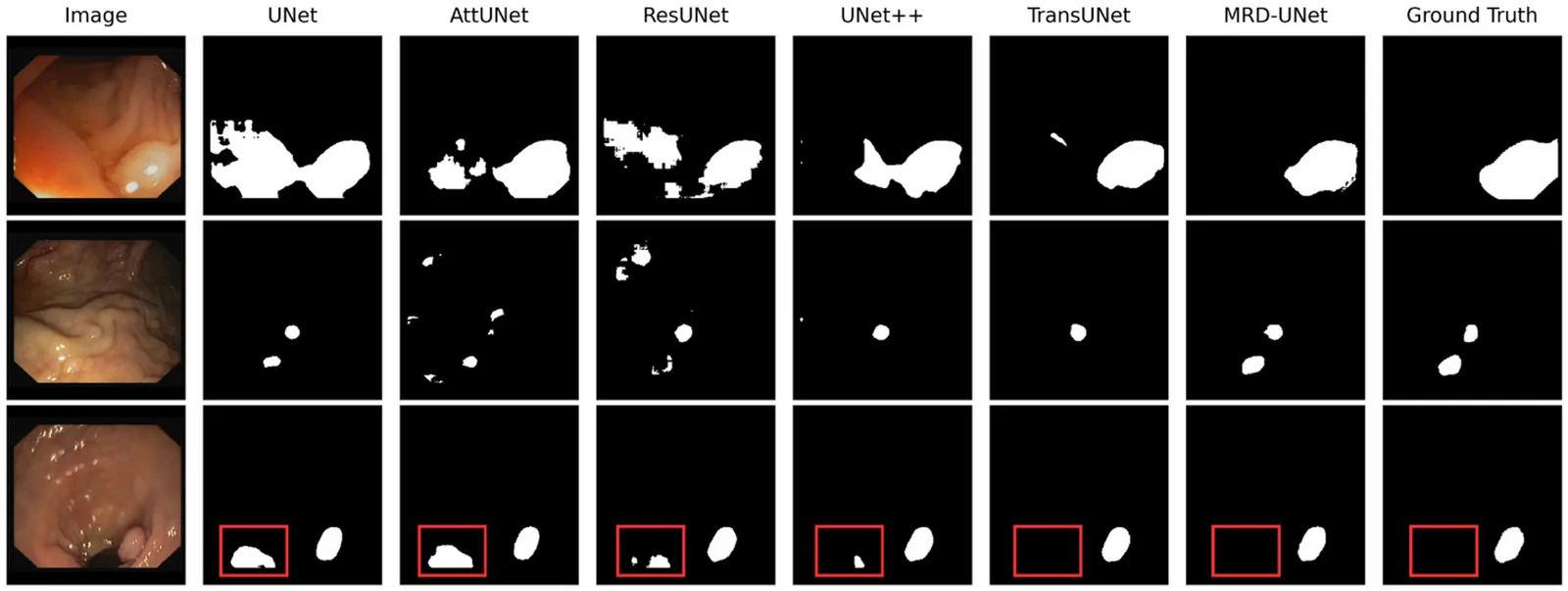
Visual comparison of segmentation results from MRD-UNet and other models on challenging samples from the CVC-ClinicDB task

**Fig. 5:**
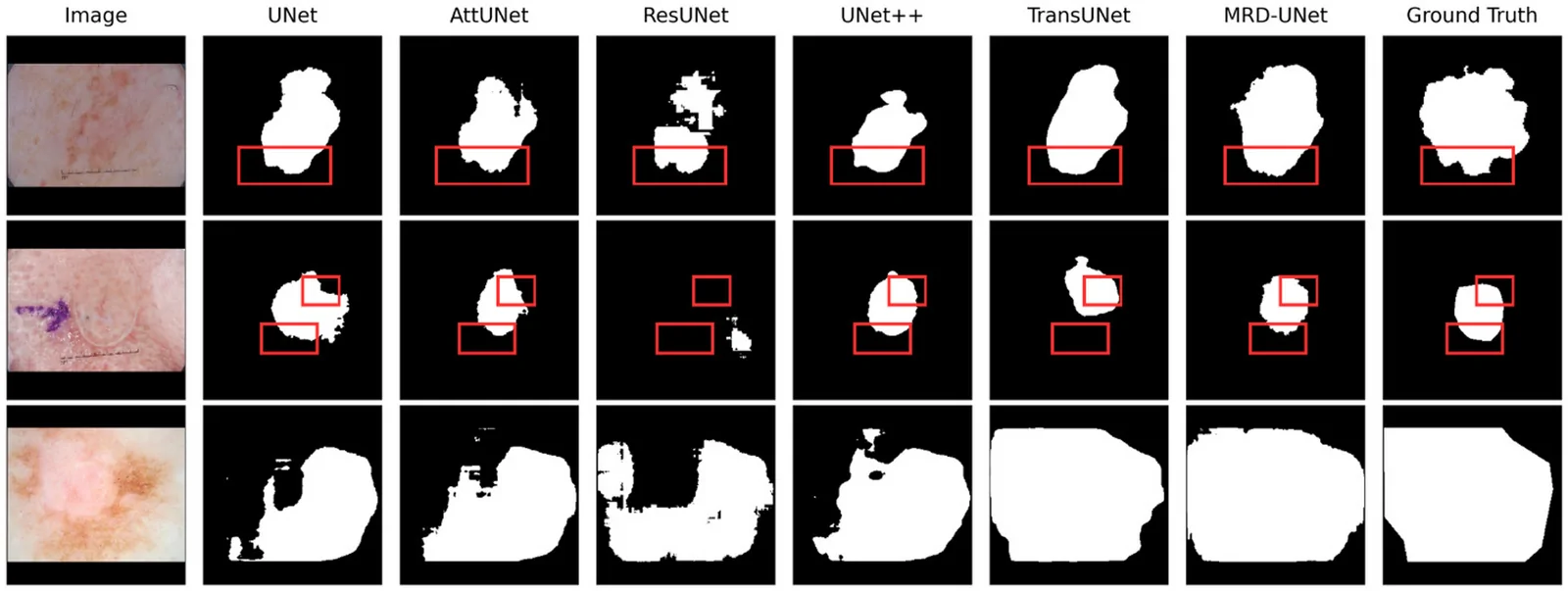
Visual comparison of segmentation results from MRD-UNet and other models on challenging samples from the ISIC-2018 task

The qualitative analysis presented in Figs. 4 and 5, featuring examples selected from challenging cases, clearly shows that MRD-UNet captures boundary details more effectively and exhibits improved spatial consistency compared with baseline methods. For clearer visual comparison, a red box was drawn at identical coordinates across all predictions. In Fig. 4, particularly under low illumination and heterogeneous tissue conditions, UNet, AttUNet, and ResUNet tend to produce false positive regions or miss smaller polyp instances, whereas MRD-UNet preserves structural integrity and delineates polyp boundaries more accurately. In Fig. 5, images with complex hair artifacts and ambiguous lesion boundaries pose serious challenges especially for UNet and ResUNet, which frequently yield over-segmented or fragmented masks; the proposed model achieves more coherent boundary delineation in these cases. These observations are consistent with the HD95 scores reported in Tables 2 and 3. 

**Fig. 6:**
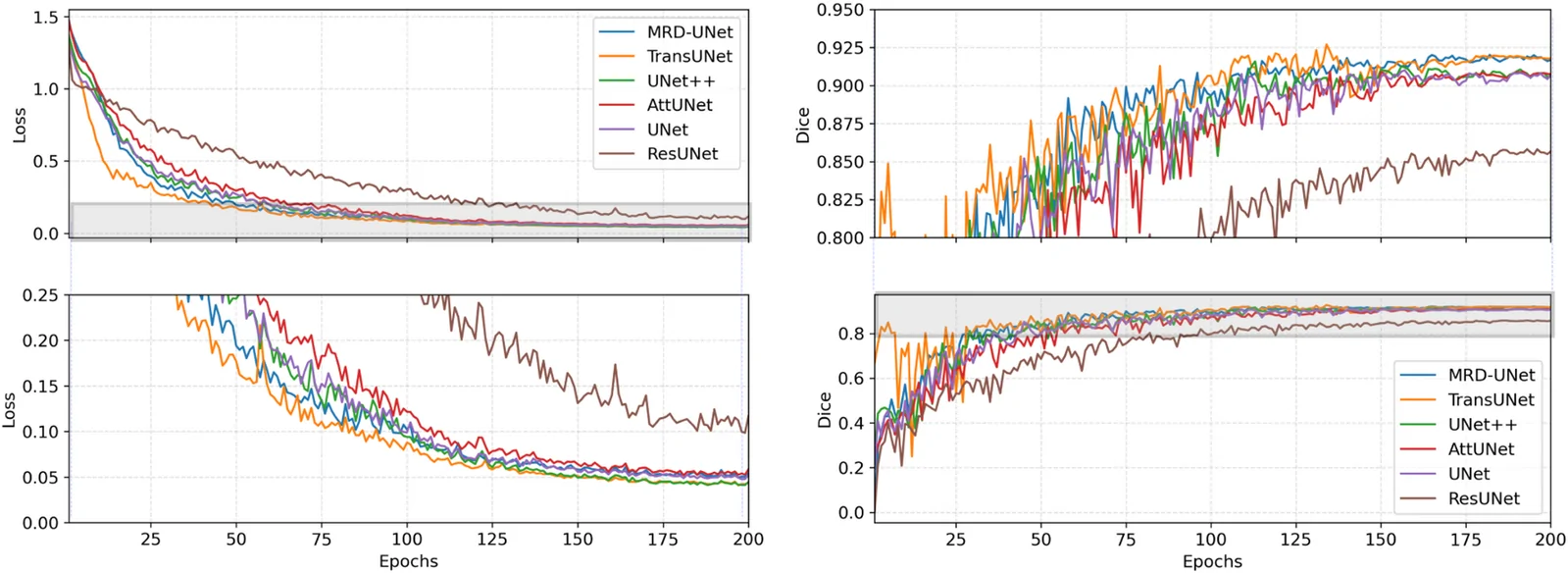
Visualization of training trends for segmentation networks on the CVC-ClinicDB task. The plots show (left) training loss and (right) validation DSC over 200 epochs, with zoomed-in views provided below each plot for detailed comparison

**Fig. 7:**
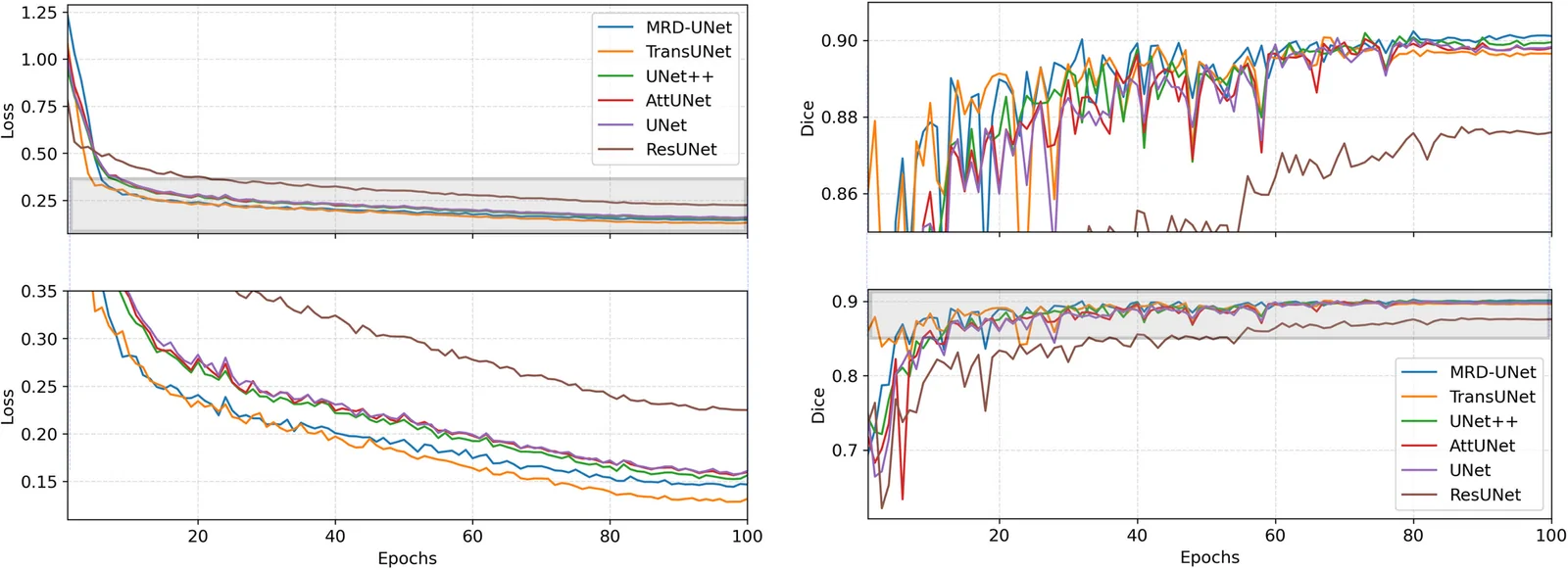
Visualization of training trends for segmentation networks on the ISIC-2018 task. The plots show (left) training loss and (right) validation DSC over 100 epochs, with zoomed-in views provided below each plot for detailed comparison

Figures 6 and 7 present the training loss and validation DSC curves for MRD-UNet and the compared models on CVC-ClinicDB and ISIC-2018, respectively. On CVC-ClinicDB (Fig. 6), MRD-UNet achieves competitive loss reduction from the early epochs and maintains stable convergence throughout training. ResUNet exhibits notably higher loss values and slower convergence compared to all other models. In terms of validation DSC, MRD-UNet and TransUNet reach the highest values by the end of training, with MRD-UNet showing a smoother convergence trajectory in the later epochs. On ISIC-2018 (Fig. 7), the models converge more uniformly across 100 epochs. MRD-UNet attains one of the highest final validation DSC values alongside TransUNet, while ResUNet again shows slower improvement. Overall, the training dynamics are consistent with the quantitative results reported in Tables 2 and 3.

## Discussion

In this study, we developed the MRD-UNet architecture to achieve a balance between segmentation accuracy and computational efficiency in medical imaging. The model is built upon two key components: a multi-scale residual module (MDF) designed to effectively capture multi-scale features, and attention mechanisms particularly using coordinate attention (CA) to highlight and refine salient regions in feature maps. Our ablation studies demonstrate the contributions of each component. Removing the MDF blocks and replacing them with standard residual blocks led to a notable performance drop on both datasets (Tables 7 and 8). Removing CBAM yielded a marginal DSC increase on CVC-ClinicDB (0.9203 vs. 0.9200) but a notable decrease on ISIC-2018 (0.9079 vs. 0.9128), suggesting that bottleneck attention is more critical for dermoscopic lesion segmentation where inter-class variation is higher.

In terms of overall performance, MRD-UNet outperforms all CNN-based baselines (UNet, ResUNet, AttUNet, UNet++) on both CVC-ClinicDB and ISIC-2018. Models such as TransUNet, DA-TransUNet, and PraNet achieve higher DSC scores on CVC-ClinicDB, partly owing to their pretrained encoders or reverse attention mechanisms; nevertheless, MRD-UNet produces competitive results under from-scratch training conditions while requiring approximately 5× fewer parameters (20.76 M vs. 108 M for DA-TransUNet). Among models trained without pretrained encoders, MRD-UNet achieves the lowest HD95 on CVC-ClinicDB (5.14), indicating superior boundary delineation compared to CNN-based counterparts. On ISIC-2018, MRD-UNet achieves the highest DSC among all evaluated models (0.9106). Despite its long-range dependency modeling capability, SSM-based U-Mamba is outperformed by MRD-UNet on both CVC-ClinicDB (0.9140 vs. 0.9013) and ISIC-2018 (0.9106 vs. 0.9067). The computational efficiency analysis (Table 4) shows that MRD-UNet requires substantially fewer parameters and achieves higher FPS than transformer-based methods without a proportional loss in accuracy.

To assess generalization, all models were trained on ISIC-2018 and evaluated directly on the PH2 dataset without fine-tuning. MRD-UNet-S achieved the highest DSC (0.9146) among all evaluated models under this cross-dataset setting, outperforming all models including U-Mamba (0.9143), DA-TransUNet (0.9120), and TransUNet (0.9119). MRD-UNet also demonstrates competitive cross-dataset performance (DSC: 0.9136) (Table 10). Several models that achieve similar DSC scores on ISIC-2018 show more pronounced differences under cross-dataset evaluation, suggesting that PH2 provides a more discriminative benchmark for assessing generalization capacity. However, as PH2 shares the same imaging modality with ISIC-2018, broader generalization claims would require validation on datasets with different acquisition conditions.

The model also presents certain limitations. The integration of multi-scale dilated processing and directional attention introduces a computational overhead relative to simpler architectures, and the model can still produce small segmentation errors in difficult scenarios as illustrated in Figs. 4 and 5.

## Conclusions

In this work, we introduce MRD-UNet, an architecture that offers a balanced approach between accuracy and computational efficiency for medical image segmentation. We deploy MDF blocks at every encoder and decoder stage rather than only at the bottleneck; each block partitions input channels into disjoint subsets routed to branches with distinct dilation rates; and CBAM, attention gates, and coordinate attention are each placed where they address a specific representational need. Our experiments on CVC-ClinicDB and ISIC-2018 demonstrate competitive performance against both CNN-based and transformer-based models, and ablation studies confirm that each component makes a distinct contribution. Cross-dataset evaluation on PH2 without fine-tuning further indicates that the multi-scale dilated feature extraction strategy generalizes across datasets within the same imaging modality, with MRD-UNet-S achieving the highest DSC among all evaluated models despite only 5.20 M parameters. Nonetheless, the computational overhead introduced by multi-scale processing results in lower FPS compared to simpler architectures, which may be a constraint in real-time clinical settings. Future work will focus on extending evaluation to diverse imaging modalities and exploring model compression techniques to improve the FPS–accuracy trade-off.

## Data Availability

The data supporting the findings of this study are publicly available. The datasets were sourced from the following locations: CVC-ClinicDB dataset (https://polyp.grand-challenge.org/CVCClinicDB/), ISIC-2018 dataset (https://challenge.isic-archive.com/data/#2018/), PH2 dataset (https://www.fc.up.pt/addi/ph2%20database.html).

## Abbreviations

AG: Attention gates
BCE: Binary cross-entropy
CA: Coordinate attention
CBAM: Convolutional block attention module
CNN: Convolutional neural networks
DSC: Dice similarity coefficient
FCN: Fully convolutional networks
FLOPs: Floating-point operations per second
FPS: Frames per second
IoU: Intersection over union
MDF: Multi-path dilated fusion
MRD-UNet: Multi-attention residual dilated U-Net model
